# Medical Opinion and Movement

**Published:** 1909-04-24

**Authors:** 


### Medical Opinion and Movement.

THE Causes of Abortion are many and varied, but the proportion of cases of this lesion in which no one of them can be detected is still very considerable. An American author, Dr. Davis, of Philadelphia, claims to have discovered that motoring is a potent agent in the production of separation and expulsion of the fertilised ovum from the uterus. He explains this sequence by supposing that the movement of the car subjects the patient to a rapid succession of numerous small jars, and alludes to the facts that, whereas some pregnant women sustain severe accidents or surgical operations without aborting, others may find the use of a sewing-machine pedal prevents the proper continuance of pregnancy. But unless he proposes to elevate this into a general principle, the reasoning is by no means ideal; and to lay down a rule that a succession of minute shocks disposes to abortion more than do single severe ones is a proposition that will take a good deal of proving. As far as that goes, the pedal question is by no means settled beyond dispute. Be that as it may, the author narrates but two cases in support of his contentions, neither of which is convincing. On this slender substratum he raises an edifice of dogma which includes a special symptomatology and ætiology of motor-car abortion. He regards motoring as distinctly dangerous in pregnancy, except towards the latter months; but he seems to realise that not many motor-owning patients will accept his opinions or follow his advice.

THE Intractable Vomiting of Pregnancy is one of those exasperating conditions, which every medical man has to treat sooner or later, wherein every resource of the therapeutist will sometimes fail. A very unusual variety is described in *La Tribune Medicale*, in which this symptom appeared in a tabetic woman, and is regarded as due to gastric crises. The patient was thirty-five years of age, pregnant for the third time, and subject to attacks of abdominal pain lasting about four days, during which nothing whatever could be retained in the stomach. During the intervals, which were of nearly equal duration, she vomited occasionally, but not in a way that could be called pernicious or intractable. As for the tabetic condition, she presented Argyll-Robertson pupils, diminished but not abolished knee-jerks, a moderate degree of anæsthesia of the right leg, and a slight lymphocytosis of the cerebro-spinal fluid. No indisputable history of syphilis could be obtained. In two previous pregnancies—the offspring of which both died soon after birth—there was also very severe vomiting, necessitating rectal feeding and saline infusion. It must be added that for some time before the third pregnancy she had been subject to gastric crises at intervals of two or three months, and frequently coincident with menstruation. The conclusion seems reasonable that the tabetic state had also something to do with the severe crises during the period of gestation.

BEFORE the New York Obstetrical Society have been read two papers on "Movable Kidney," which are printed in the *American Journal of Obstetrics*. Dr. Ely considered chiefly the relations of this condition to other diseases, especially in the pelvis: perhaps the most interesting and original of his remarks were on the subject of appendicectomy, which operation he has on thirty-two occasions observed to be followed by entire cessation of symptoms previously attributed to the movable right kidney. The improvement noted has been, he thinks, greater than can be accounted for by the necessary rest in bed and abdominal belts ordered. Dr. Baldwin has collected the opinions of many eminent surgeons on the question of operative interference for the condition. Though great diversities of opinion and practice are revealed, it is evident that the majority are in accord with Dr. Baldwin's own convictions. These are that no case should receive operative treatment until every other means has had the most thorough trial: that many of the symptoms ascribed to movable kidney are not in fact due to it at all: that mobility of less degree than the length of the organ itself practically never causes symptoms: and that nephropexy for movable (as opposed to actually wandering) kidney is often unsuccessful.

THE current issue of the *Journal of the Royal Army Medical Corps* is exceptionally full of noteworthy contributions. Captain Bourke describes an extensive epidemic in a regiment at Bloemfontein of a disease which more resembled, he thinks, Dr. Duke's celebrated "fourth disease" than it did either scarlet fever or rubella. In all thirty-three cases occurred among the soldiers, with one death; and three among the children. Among the peculiar features were the comparative mildness of the disease, especially as regards pyrexia; several of the patients had a normal temperature throughout. An initial sore throat was the rule, but headache and vomiting were noticed only three times prior to the eruption, though the former was sometimes present after the rash appeared. A low pulse-rate was a marked feature in almost every patient. The rash was usually the first symptom, and it appeared most often on the neck and chest, spreading afterwards to the abdomen, thighs, and groins. It was distinctly scarlatiniform, but did not affect the face; and the circumoral pallor so constantly seen in true scarlet fever was conspicuously absent. Desquamation was the rule. The typical strawberry tongue of scarlet fever was seen only four times: the posterior cervical and the inguinal glands were always enlarged. It so happened that in many cases the incubation periods could be fairly accurately determined, and they were found to vary between seven and twenty-eight days, most often about fifteen: this of itself is almost enough to exclude scarlet fever as the cause of the outbreak. Five cases of albuminuria were noted, and three of otorrhœa: no other complications occurred. Captain Bourke attributes the epidemic to contagion conveyed by infected blankets. About twenty-one days after the issue of one extra blanket per man just before the approach of the cold weather the first cases appeared: these blankets were not new, but had been in store for one to two years since their last usage.

Major C. G. Spencer records a case of Sarcoma treated with Coley's fluid. The patient was first seen in 1906 with a hard swelling reaching from the pubes nearly to the umbilicus, and complaining of frequent and painful micturition. The urine contained a little albumin, but no blood nor casts. In September of that year an attempt was made to remove this tumour, which was shown to be a sarcoma by microscopical examination; but as it infiltrated the pubes, the recti, and the bladder, the operation was abandoned. Twelve injections of Coley's fluid were then given, during which the general state deteriorated greatly: they were then stopped and the patient sent away on sick furlough, from which he returned with a gain of fifteen pounds in weight and a diminished tumour. At the beginning of 1907 he had another course of injections, attended by the same result of cachexia and tenderness of the growth, but succeeded by rapid improvement after the end of the series. A third set of nineteen injections was then ordered in April 1907; and in December 1908 he was perfectly well, with no sign of any tumour, and no vesical symptoms. Major Gunter publishes some notes on surgical technique which are of great importance. He has experimented with various methods with a view to the selection of those which can be carried out as well in the field as in a hospital: thus he has used empty biscuit tins instead of drums for sterilising dressings. As a result of painstaking bacteriological work he concludes that the use of gloves is a desirable part of operative technique, but that guards for the mouth and the field of operation are unnecessary. He thinks highly of the iodine method of preparation of the skin after simple dry shaving, described already in The Hospital, January 23, 1909; p. 426. The advantages of this easy procedure in military surgery are obvious.

An interesting case, in which Gall-Stones were found in the Urinary Bladder of a woman, is published by Michel in the *Zentralblatt f. Gynæk.* Three years ago the patient had a definite hepatic crisis, accompanied by symptoms of peritonitis in the right half of the abdomen, especially localised to the left hypogastrium Her attendant diagnosed a perityphlitic effusion, secondary to a cholecystitis. So far as could be judged this effusion was slowly absorbed, but the patient suffered from time to time with suppression of urine and vesical pains. A year ago the symptoms referred to the region of the bladder reappeared, and topical irrigations were prescribed as though for a cystitis. This proved of no avail, and eventually the diagnosis of vesical calculus was made, and the patient was sent to Dr. Michel for operation. Cystotomy was performed by him, per vaginam, and four stones were discovered embedded in the bladder wall. These were removed with difficulty, and the bladder and vagina were then sewn up with catgut. The stones proved to be biliary calculi, the two largest weighing respectively 11.4 and 10.1 grammes. It is likely that a direct communication existed between the gall-bladder and the urinary bladder, for bile was at times discovered in the urine, both before and after operation, and at no time was there the slightest evidence of jaundice. The author's surmise is that, as a consequence of the original inflammatory process, a communication was formed between the gall-bladder and the right ureter, down which the gall-stones travelled into the bladder. This bilio-urinary fistula has remained patent.

Cocci, first assistant to Professor Burci at the surgical clinic at Florence, contributes to *Il Morgagni* an interesting study on the results of Partial Prostatectomy. The surgical treatment of hypertrophy of the prostate, according to the author, falls into three well-marked divisions. In the first are such cases in which methodical catheterisation is sufficient, aided, if necessary, by suprapubic vesical fistula. In the second class fall such cases as are benefited by palliative operative treatment, under which Cocci would understand White's operation, vasectomy, and possibly also Bier's ligation method, while in the third class are included such cases as demand more radical and severe measures, such as prostatotomy, partial prostatectomy, and complete enucleation. Discussing these various classes, Cocci expresses the opinion that in the first methodical catheterisation is the method of choice, while the formation of a suprapubic fistula is to be deprecated on the ground that it is really useless and at the same time open to grave objection on various counts. He is disinclined to attach much importance to palliative operations of the Bier-White class, as statistics have not shown that they are efficacious. This point, however, he does not dwell upon, and he leaves his reader in ignorance of the statistics which he relies on. It is highly important that this question of the uselessness or otherwise of vasectomy should again be considered. English surgeons are more or less united in condemning the operation, but hitherto no reliable comparative statistics have been adduced to prove that those who still rely on the operation, as Rovsing of Copenhagen and other Continental authorities do, are wrong.

Turning to the radical operations Cocci expresses himself greatly in favour of partial prostatectomy in selected cases. Prostatotomy he dismisses as an operation that has few points in its favour and many against it, while total prostatectomy, which is the ideal operation in most cases, is out of the question in some instances where the patients cannot stand such prolonged and serious interference. The author gives a very interesting account of the evolution of the partial method, from which it appears that Amussat, as early as in 1827, attempted it by the hypogastric route. Dittel in 1885 first seriously attempted the operation, and he was followed by Belfield in America, Guyon in

France, and many others. Since total enucleation has become popular, through the exertions and the able work of Freyer, the partial operation has fallen into disfavour, as the author thinks, unjustifiably. He sums up the points in favour of the operation as follows: it interferes less with the normal relations of the structures attacked; it permits the surgeon to remove the obstruction without opening up artificial pouches at the base of the bladder where pus and blood may stagnate; it is a less severe operation than total enucleation. This summary is undoubtedly too favourable, and the excellent results established in the four cases which the author adduces can hardly be accepted as conclusive evidence in favour of the operation. Where in any case there is a possibility of radically removing the gland the surgeon will find Freyer's statistics and results too convincing to allow him to resort to the partial operation, which has apparently as many dangers as the total operation, and which can scarcely be said to be a radical operation in the true sense of the word.

---

In an interesting paper read before the Belgian Academy of Medicine Dr. Desguin discusses the differential diagnosis between Typhoid Fever and the gastro-intestinal type of Pneumococcal Infection, and he demonstrates several practical means by which a correct diagnosis may be attained. A colorimetric examination of the blood, using Tallqvist's scale, is one of the simplest methods at the disposal of the practitioner. In typhoid fever the hæmoglobin content may fall to 70 or 60 per cent., although the appearance of the patient may not suggest such a condition. On the other hand, in a pneumococcal infection the patient is pale and oligohæmic; but the hæmoglobin content is the same as is a healthy person. A second means consists in estimating approximately the relative proportion of red and white corpuscles. In typhoid there is leucopenia; in pneumonia hyperleucocytosis. The author maintains that these two signs never fail, and are quite convincing. As a third means of diagnosis, however, he suggests the inoculation of white mice with a few drops of the patient's blood. This animal is extremely sensitive to the pneumococcus, but is little affected by Eberth's bacillus. In the former case the mouse becomes rapidly ill. If the animal is then killed and the blood from the heart examined bacteriologically, the presence of the pneumococcus is easily testified. But if at the end of 24 hours the mouse remains healthy, it may be concluded that the patient is suffering from typhoid. The author is of opinion, however, that only in exceptional cases is it necessary to have recourse to this third test, as the first two are quite sufficient for all practical purposes.

---

It is a well-recognised fact that lactation causes more or less complete suppression of menstruation, so that the activity of the mammary gland is evidently in some way antagonistic to the utero-ovarian functions. This fact suggested to Pryor, of New York, and Schrober, of Philadelphia, in 1901, the treatment of uterine fibroid and uterine hæmorrhage by Mammary Oportherapy. Dr. Batuaud, in a contribution to the *Journal de Médecine*, discusses this method of treatment. He is unable to confirm the statements of these American authors in regard to any diminution in size of uterine fibroids as a result of the treatment, but he finds that the administration of mammary gland dried and powdered has a pronounced effect on the hæmorrhage from fibroids, and in cases of abnormal menstruation, too frequent, too profuse, or of too long duration, it is able to bring about a return to the normal condition. Frequently the effect is immediate, but in about 5 per cent. of cases the treatment fails entirely. The dry powder of mammary gland is given in cachets of 5 centigrammes corresponding to 3.5 grammes of the fresh gland. The usual dose is two cachets taken before meals every day, and this may be doubled during hæmorrhage or menstruation. Intervals of several days may be made in the course of the treatment, according to the effects produced.

---

In a contribution to the *International Journal of Surgery*, Dr. Walter L. Hunt discusses the treatment of fracture of the neck of the femur. By a brief survey of the literature on the subject and of the opinions expressed by several leading surgeons, he shows that the idea is very prevalent that bony union almost invariably fails to take place, and that satisfactory treatment of these cases is extremely difficult. He would maintain, however, that this failure is due to faulty technique, and proceeds to demonstrate the method of Professor Maxwell, by which both the author and Dr. C. E. Ruth, of Iowa, have succeeded in obtaining most gratifying results. As this method appears to be little known, a short account of it may prove useful. The patient is put on his back. The thigh is flexed on the abdomen to relax the psoas and iliacus muscles and bring their line of action above the neck of the femur and prevent their forcing soft tissue between the fragments. Extension is then made, and at the same time an assistant makes traction outwards and raises the great trochanter to the same level as its fellow. Extension on the leg is kept up by means of adhesive strapping extending well above the knee, and a five to twenty-five pound weight over a pulley. Binder's board is now moulded to the internal, anterior, and posterior aspects of the upper part of the thigh, and around this a broad band of muslin is extended by a cord over a pulley at the side of the bed, to which is attached a weight of eight to sixteen pounds. This pulley must be at such a height and carry sufficient weight to overcome the tendency to eversion and carry the trochanter to the level of its fellow on the opposite side. To effect this the direction of the pull should be outwards, ten degrees upwards towards the armpit and forwards at an angle of about forty-five degrees. The knee should be bent every four or five days to prevent ankylosis. Counter extension is made by raising the foot of the bed and also the side corresponding to the injured limb. The patient can be raised to the sitting posture daily without disturbing the fragments, and the method is said to be quite free from pain or discomfort. Four to six weeks' confinement are sufficient to procure union.

